# Trichohepatoenteric Syndrome: A Report of Two Children From Bahrain With a Novel Mutation and a Literature Review

**DOI:** 10.7759/cureus.75687

**Published:** 2024-12-14

**Authors:** Hasan M Isa, Wafa M Matar, Ghufran J Ali, Maryam Y Busehail, Narjis A Alsheala, Eman S Shajira

**Affiliations:** 1 Department of Pediatrics, Arabian Gulf University, Manama, BHR; 2 Department of Pediatrics, Salmaniya Medical Complex, Manama, BHR; 3 Department of Pediatrics, Military Hospital, Bahrain Defence Force Royal Medical Services, Riffa, BHR

**Keywords:** bahrain, dysmorphic features, failure to thrive, intractable diarrhea of infancy, poor weight gain, trichohepatoenteric syndrome

## Abstract

Trichohepatoenteric syndrome (THES) is a rare genetic disorder inherited in an autosomal recessive manner. THES primarily leads to neonatal enteropathy, typically manifesting as severe, persistent diarrhea, distinctive facial features such as frontal bossing and a broad flat nasal bridge, woolly and fragile hair, immunodeficiency resulting in recurrent infections, failure to thrive (FTT), and liver complications including fibrosis or cirrhosis. This multisystem disorder is linked to mutations in the tetratricopeptide repeat domain 37 (TTC37) gene, also known as superkiller complex (SKIC) protein 3, responsible for THES type 1, and the Ski2-like ribonucleic acid (RNA) helicase (SKIV2L) gene, also known as SKIC2, responsible for THES type 2. This case report describes two far-related pediatric patients from Bahrain diagnosed with THES type 1. Although both patients exhibited typical symptoms of this syndrome, the first patient had more severe symptoms. Diagnostic genetic evaluations confirmed THES. Patient 1 had three homozygous variants in the TTC37 gene, while Patient 2 had two variants in the same gene. Both of our patients had a novel homozygous variant. The treatment focused on supportive care and infection management. Despite these efforts, patients did not reach their growth potential. This report underscores the necessity for early identification of THES to facilitate appropriate management and genetic counseling.

## Introduction

Trichohepatoenteric syndrome (THES), also known as syndromic diarrhea, is a rare genetic disorder inherited in an autosomal recessive pattern due to mutations in the Ski2-like ribonucleic acid (RNA) helicase (SKIV2L) gene, also known as superkiller complex 2 (SKIC2) (OMIM # 614602) and the tetratricopeptide repeat domain 37 (TTC37) (SKIC3) (OMIM # 222470) genes [1,2]. Pathophysiologically, these mutations may disrupt messenger ribonucleic acid (mRNA) processing, impacting intestinal barrier functions and liver metabolism, and causing immune dysregulation [1].

The global prevalence of THES is estimated at one in 1,000,000 and is often associated with consanguinity [1,2]. It was first described by Stankler et al. [3] in 1982, following the observation of two siblings with failure to thrive (FTT), characteristic facial features, fine, woolly hair, and liver fibrosis. Their diarrhea presented early in the third week of life and resulted in death. Subsequently, Girault et al. [4] reported eight additional cases in 1994, detailing further clinical symptoms such as dermal manifestations, recurrent infections, and intellectual disability.

Patients with THES can have antenatal signs such as intrauterine growth restriction (IUGR) [1,2]. The condition typically presents with early-onset severe diarrhea, a hallmark symptom in nearly all cases, followed by FTT [1,2,4]. Other consistent features include hypertelorism, fine, woolly hair, and facial dysmorphism [1,2,4]. Patients often experience recurrent infections, liver dysfunction, and cognitive impairment, while some present with skin manifestations like café au lait spots [1,2]. Rarely, thymus atrophy, hypothyroidism, and inguinal hernias may occur, though these may be coincidental [1,2]. Owing to the multisystem involvement, genetic testing is needed to confirm the diagnosis of this syndrome, while the management is mainly supportive [2]. Despite its rarity, cases of THES have been reported in the Gulf region, though none in Bahrain. This report presents the first two patients with three mutations indicating THES in the Kingdom of Bahrain; one mutation was novel, highlighting characteristic clinical and genetic features of this rare syndrome.

## Case presentation

Case 1

**Figure 1:**
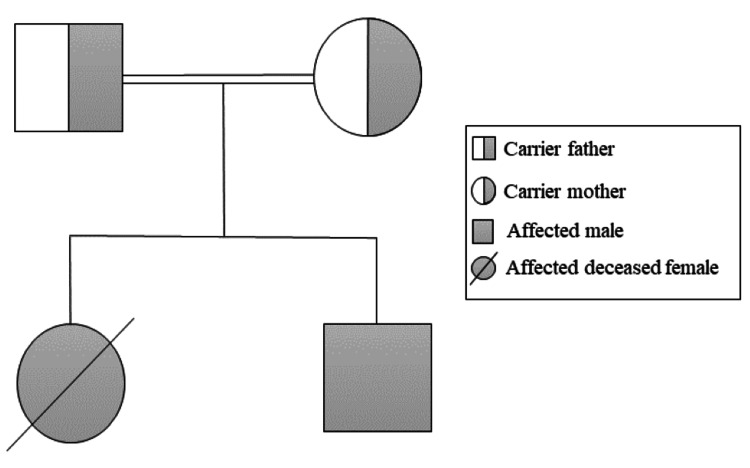
Family pedigree of a one-year-old child with trichohepatoenteric syndrome type 1 (Patient 1) Image credits: Hasan M. Isa, Wafa M. Matar, and Ghufran J. Ali

This is a 14-month-old male patient born via elective cesarean section at 36 weeks of gestation due to IUGR, with a birth weight of 1.755 kilograms (kg) which required a neonatal intensive care unit (NICU) admission. The patient has consanguineous parents, and a family history of infant death (his elder sister) attributed to chronic diarrhea and renal tubular acidosis type 1. The family pedigree is shown in Figure 1.

**Figure 2:**
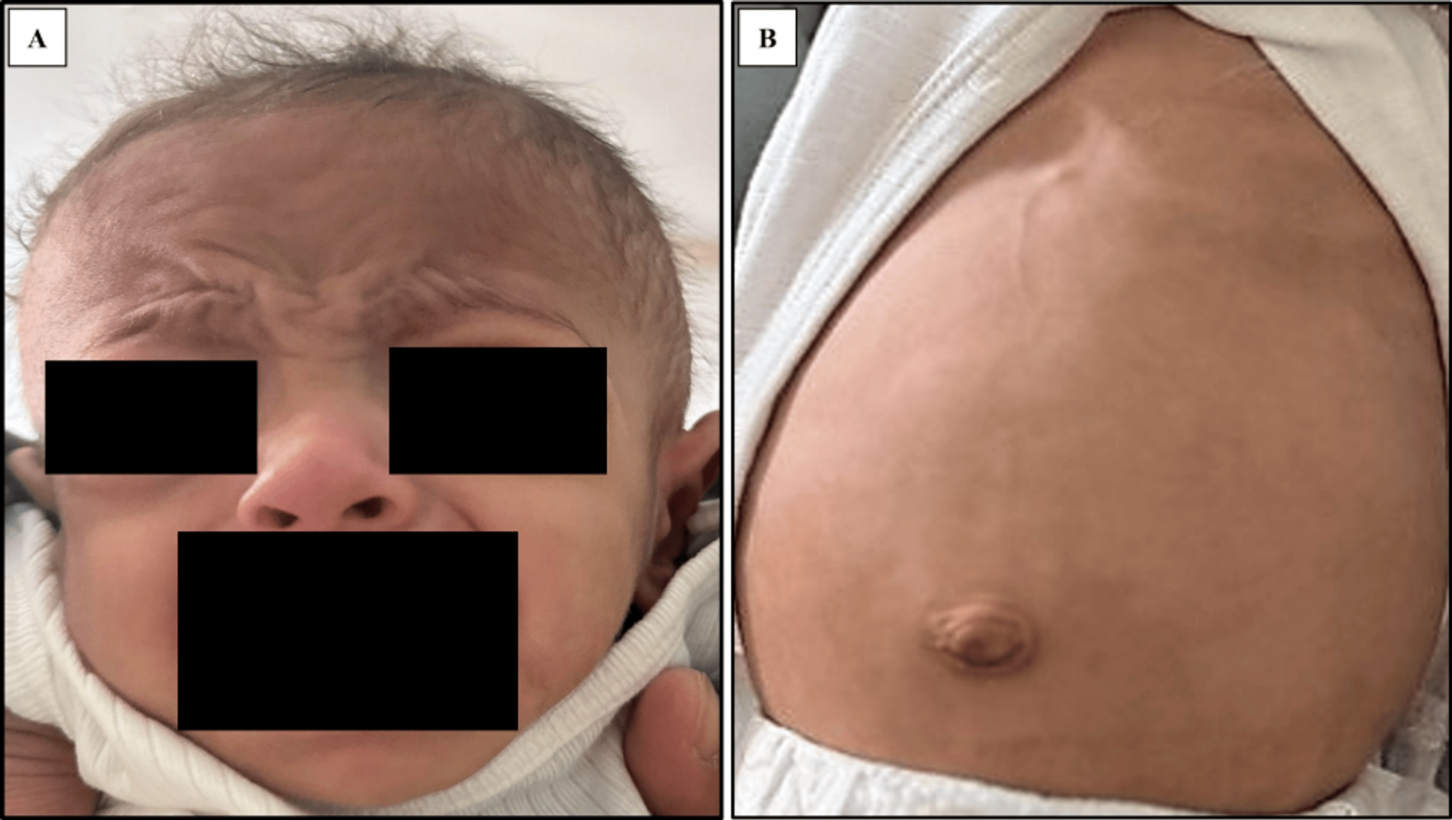
Patient 1 with trichohepatoentric syndrome The patient's pictures show dysmorphic facial features in the form of a large forehead and broad nasal bridge with sparse hair (A) and abdominal distention (B)

At the age of three months, the patient was hospitalized for severe, foul-smelling diarrhea lasting for 19 days following left inguinal hernia repair, accompanied by poor weight gain. Physical examination revealed a pale, marasmic infant with tiny, scattered café au lait spots in his legs and trunk, dry skin, and reducible right inguinal hernia. The patient also had dysmorphic facial features in the form of large forehead, broad nasal bridge, and sparse hair (Figure 2A), along with hepatomegaly and abdominal distention (Figure 2B).

Laboratory workup was performed, and the results are further detailed in Table 1.

**Table 1:** Laboratory findings of patients with trichohepatoenteric syndrome type 1 WBC: white blood cells; CD: cluster of differentiation; NR: no record; ALP: alkaline phosphatase; ALT: alanine aminotransferase; AST: aspartate aminotransferase; GGT: gamma-glutamyl transferase; Ig: immunoglobulin; TTC37: tetratricopeptide repeat domain 37 Data are presented as numbers alone or numbers (%)

Laboratory marker	Reference range	Patient 1	Patient 2
Hemoglobin (g/dL)	12.0-14.5	8.9	11.2
Platelets count (×10^9^/L)	150-400	135	981
WBC (×10^9^/L)	3.6-9.6	23.2	20.0
CD4^+^ T-helper lymphocytes absolute count (cells/µL) (%)	1800-4000 (35%-56%)	4204 (31.5%)	4027 (39.0%)
CD8^+^ T-cytotoxic lymphocytes absolute count (cells/µL) (%)	590-1600 (12%-23%)	7475 (56.0%)	4041 (39.1%)
CD19^+^ B-lymphocytes absolute count (cells/µL) (%)	430-3000 (11%-41%)	400 (3.0%)	(13.6%)
Total protein (g/L)	57-82	48	65
Serum albumin (g/L)	38‒54	34	30
Serum globulin (g/L)	15-30	14	35
Total bilirubin (µmol/L)	≤21	25	4
Direct bilirubin (µmol/L)	≤5	NR	2
Indirect bilirubin (µmol/L)	<18	NR	2
ALP (IU/L)	150-420	476	87
ALT (IU/L)	≤41	56	6
AST (IU/L)	≤40	NR	27
GGT (IU/L)	≤73	297	16
IgA (g/L)	0.10-0.50	0.28	NR
IgG (g/L)	2.40-8.80	0.47	NR
IgM (g/L)	0.20-1.00	0.74	NR
Genetic results (TTC37 gene variants)			
c.2170T>C (p.Cys724Arg)	Negative	Homozygous	Homozygous
c.4507C>T (p.Arg1503Cys)	Negative	Homozygous	Homozygous
c.4534C>T (p.Pro1512Ser)	Negative	Homozygous	Not detected

**Figure 3:**
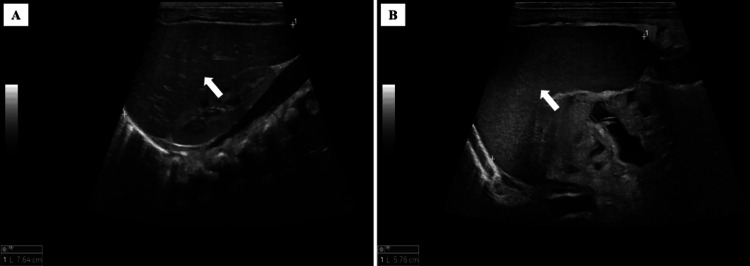
Abdominal US images of a patient with trichohepatoenteric syndrome (Patient 1) US: ultrasound (A) US image showing hepatomegaly with altered parenchymal echogenicity (arrow); (B) US image showing splenomegaly with altered parenchymal echogenicity (arrow)

Liver function tests showed low serum protein and albumin but high total bilirubin, alkaline phosphatase, alanine aminotransferase, and gamma-glutamyl transferase. Immunological tests revealed a significantly decreased cluster of differentiation (CD) 19^+^ B-cells and an increased CD8^+^ cytotoxic T-cells which was higher than CD4^+^ helper T-cell lymphocytes. Additionally, immunoglobulin (Ig) G level was low. A radiologist suggested the possibility of a genetic disorder based on an abdominal ultrasound that indicated an altered parenchymal echogenicity with hepatomegaly (Figure 3A) and splenomegaly (Figure 3B).

Genetic tests with whole-exome sequencing were subsequently performed. The patient was exclusively breastfed. Transitioning to an exclusive amino acid formula (Neocate) led to temporary improvement in his diarrhea, and the patient was discharged home with a follow-up for the genetic results.

At the age of five months, genetic analysis revealed three homozygous variants in the TTC37 gene (Table 1), associated with autosomal recessive THES type 1, prompting genetic counseling. Despite routine follow-ups, the patient faced frequent admissions for intractable diarrhea and poor weight gain. At the age of six months, the patient was hospitalized for a trial of nasogastric tube (NGT) feeding; however, abdominal distention was noted. This led to counseling the parents regarding total parenteral nutrition (TPN), but they were initially reluctant due to concerns stemming from a previous unfavorable outcome with TPN in the first child at another hospital. After further discussion and emphasizing its importance in managing the child's weight, they ultimately consented. At the age of seven months, TPN was placed, consisting of normal saline, 5% dextrose, 10% aminoplasmin, and 20% intralipid infusion via central line which improved the patient's condition and weight. However, complications arose, including a central line infection and sepsis, necessitating antibiotics (vancomycin and meropenem) and multidisciplinary care. Cardiac evaluation showed normal functions with a small atrial septal defect (ASD) < 2.5 mm but no vegetations. Moreover, the patient received intravenous immunoglobulins for recurrent infections and local antifungal treatment for nappy rash. However, the patient was discharged against medical advice upon his mother’s request. The central line was also removed. Six days following discharge, at the age of eight months, severe dehydration resulted in metabolic acidosis and prerenal azotemia, leading to pediatric intensive care unit (PICU) admission. In the PICU, the patient experienced a cardiac arrest that lasted for eight minutes but was successfully resuscitated. After stabilization, the patient was transferred to a general ward.

During a 100-day hospital stay, the patient received ongoing treatment, including antibiotics (vancomycin and meropenem) and iron supplementation (initially subcutaneous epoetin for 10 days, followed by oral iron syrup). A hematology consultation was requested for leukocytosis and transient thrombocytopenia; however, bone marrow aspiration was not suggestive of leukemia or other abnormalities. The patient also experienced an episode of respiratory desaturation, which was managed with high-flow oxygen via nasal cannula. A peripherally inserted central catheter (PICC) was reinserted by an interventional radiologist to support feeding and growth. After clinical stabilization, the PICC line was removed, and the oral calorie intake was increased from 0.86 kcal/ml to 1 kcal/ml by increasing high-energy carbohydrate (Fantomalt) from two scoops per feed to three scoops per feed of extensively hydrolyzed milk formula (Novolac Allernova) 200 ml for four times per day. Moreover, the mother was advised to continue solid food introduction as tolerated. The patient was discharged home and given outpatient appointments with multidisciplinary teams for further follow-up.

**Figure 4:**
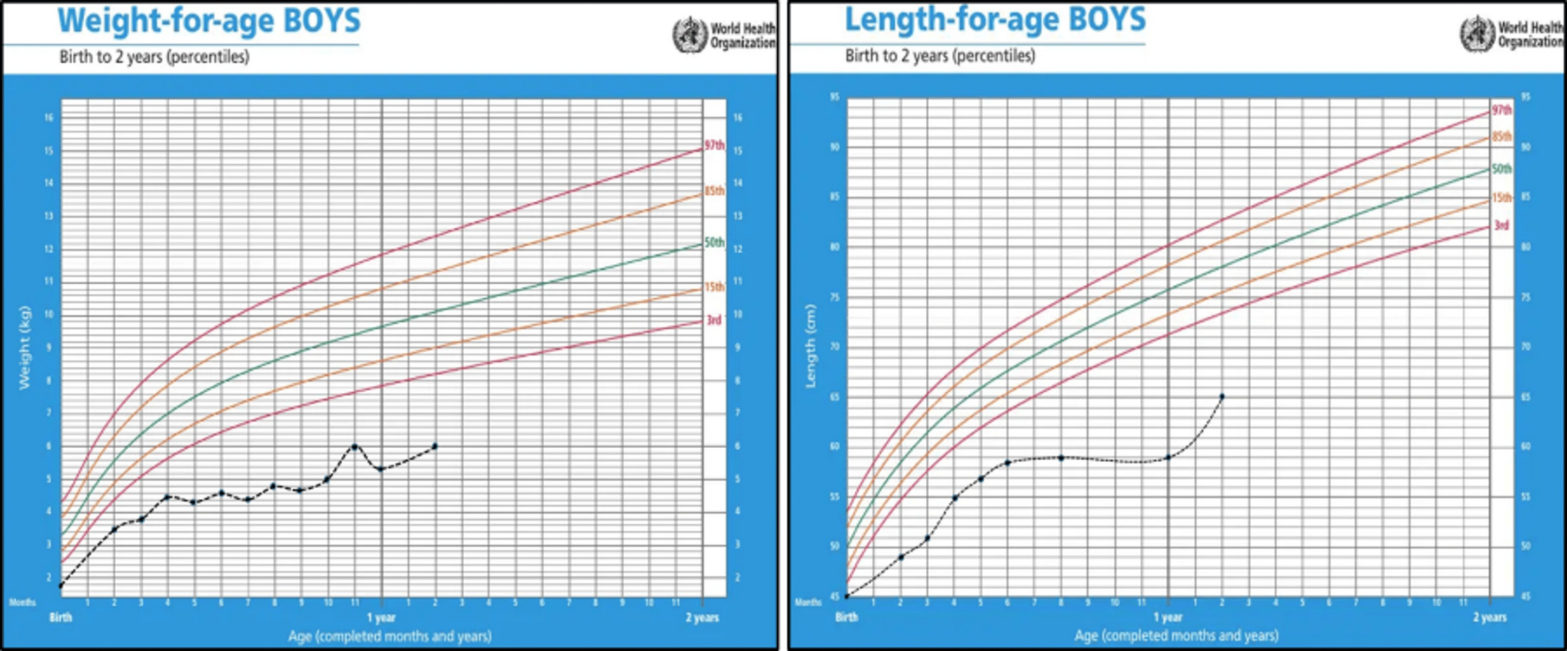
Growth chart showing the weight and length growth impairment of a one-year-old child with trichohepatoenteric syndrome type 1 (Patient 1) Both weight and length remain below the third centile for age and sex

Currently, the patient is still receiving his special milk formula and consumes more solid foods than milk under dietitian guidance. The patient’s vaccination status is up to date for his age. He weighs 6 kg and measures 65 cm, remaining below the third centile for age and sex (Figure 4).

**Figure 5:**
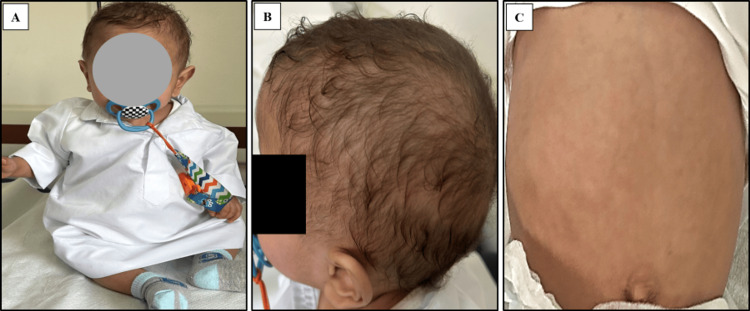
Patient 1 on follow-up visit The patient's pictures show improvement in his condition with newly acquired motor skills such as sitting (A), improvement in hair condition (B), and reduced abdominal distention (C)

The patient's clinical improvement is evident through newly acquired motor skills including clapping, waving bye-bye, and sitting (Figure 5A), enhanced hair condition (Figure 5B), and reduced abdominal distention (Figure 5C).

Ongoing multidisciplinary care includes surgical planning for right inguinal hernia and hypospadias variant repair, alongside follow-up appointments with dietitian, gastroenterology, immunology, and physiotherapy specialists.

Case 2

**Figure 6:**
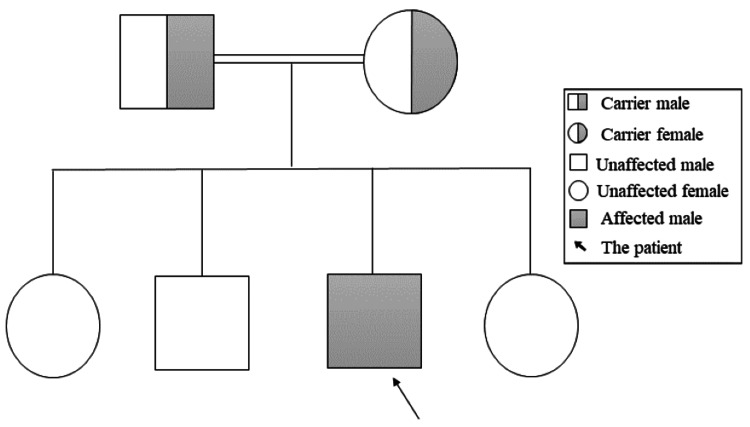
Family pedigree of a four-year-old child with trichohepatoenteric syndrome type 1 (Patient 2) Image credits: Hasan M. Isa and Ghufran J. Ali

This is a four-year-old boy, a far relative of Patient 1. The patient was born to consanguineous parents, and he has three unaffected siblings. The patient's family pedigree is shown in Figure 6.

The patient was born prematurely at 31 weeks gestational age via cesarean section due to IUGR and absent diastolic blood flow. His birth weight was 1.18 kg, and his Apgar scores were 5 and 7 at one and five minutes, respectively, indicating signs of poor respiratory efforts. Postnatally, he was immediately admitted to NICU. The patient was intubated for seven days before being weaned to nasal prongs. He also experienced a stage one retinopathy of prematurity, which resolved upon follow-up. A septic workup was performed twice and returned negative for any organisms. On day three of life in the NICU, the baby developed sudden abdominal distention accompanied by fresh blood from the NGT, which necessitated a review by the surgical team; however, he was stabilized and did not require any surgical intervention. He was monitored with serial abdominal X-rays, all of which were normal. Hemoglobin electrophoresis, glucose-6-phosphate dehydrogenase (G6PD) activity testing, and thyroid function tests were conducted and returned normal results. The patient was discharged after 42 days in the NICU with satisfactory weight gain and feeding. His vaccination status is up to date.

**Figure 7:**
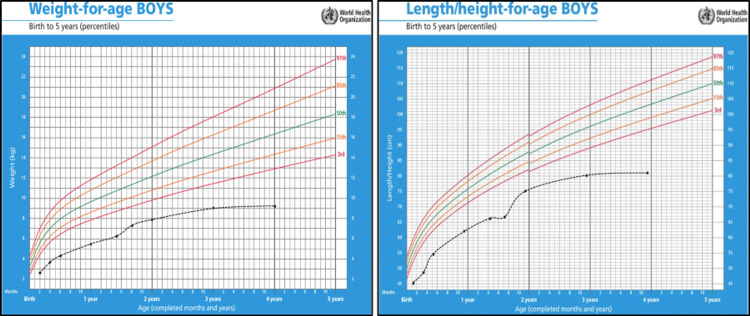
Growth chart showing the weight and length/height growth impairment of child with trichohepatoenteric syndrome type 1 (Patient 2) Both weight and length/height are below the thirdcentile for age and sex

At the age of seven months, the patient presented to the pediatric clinic with global developmental delay, FTT, and chronic diarrhea for further investigations. The patient was admitted for NGT insertion due to poor weight gain associated with inadequate oropharyngeal coordination. He was placed on a high-energy formula (Similac); then, he was discharged home. Despite these interventions, his weight and length consistently remained two standard deviations below the mean for his age and sex (Figure 7).

**Figure 8:**
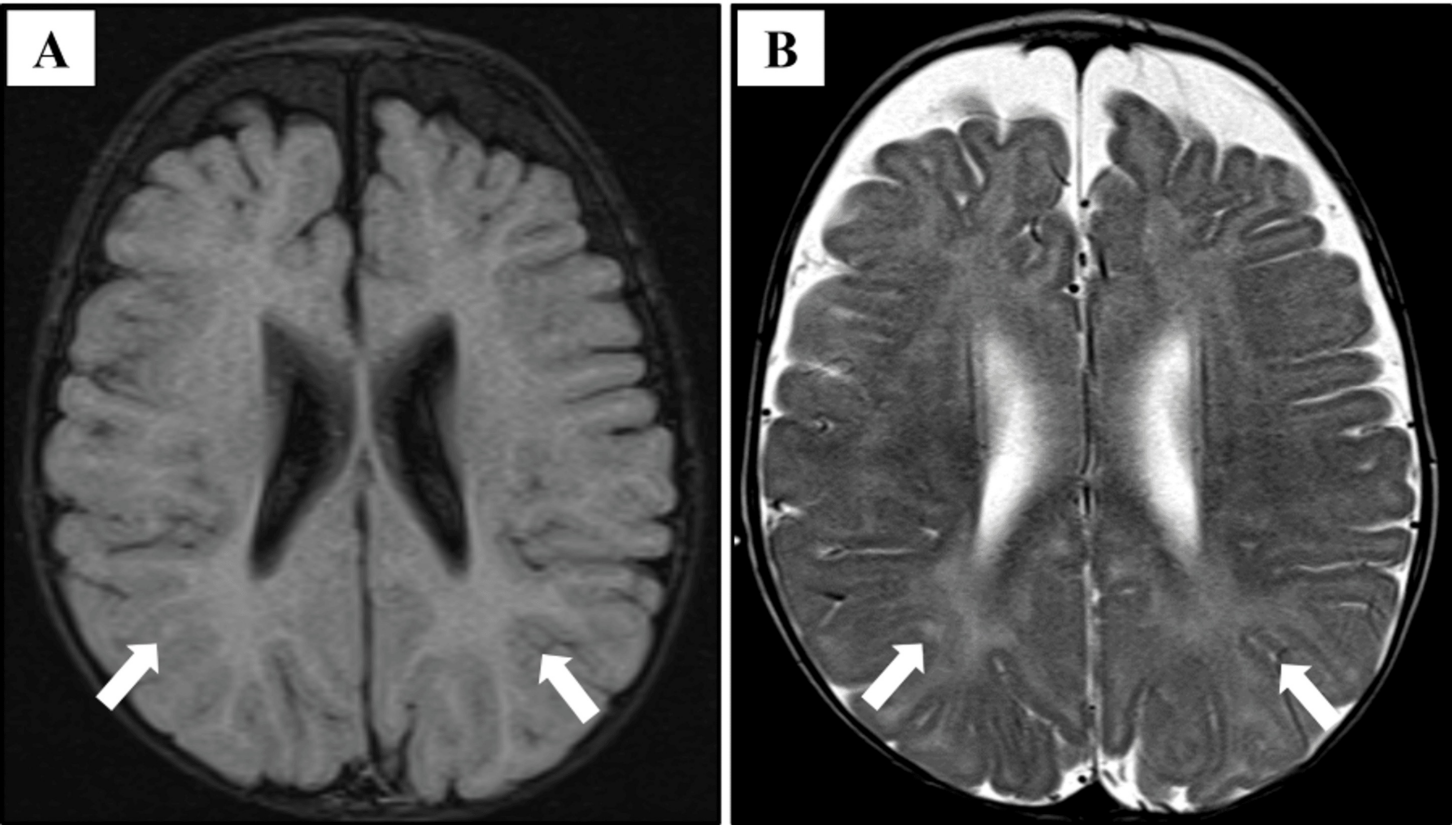
An axial brain MRI of a patient with trichohepatoenteric syndrome (Patient 2) MRI: magnetic resonance imaging (A) A T2-fluid-attenuated inversion recovery (FLAIR) MRI image showing periventricular and peritrigonal white matter hyperintensities extending to some areas of the ventricular ependymal surface (arrows). (B) A T2-weighted MRI image showing the same findings (arrows)

At the age of eight months, the patient was readmitted due to acute bronchiolitis, and he received supportive treatment as recommended. Following discharge and further follow-up, he was readmitted at nine months of age for investigations related to FTT and chronic diarrhea. During this admission, a Gastrografin meal and follow-through study were performed and reported normal results. Additionally, celiac disease was excluded. Hypotonia noticed in examination and global developmental delay necessitated a magnetic resonance imaging (MRI) which was performed at one year of age and showed periventricular and peritrigonal white matter hyperintensities extending to some areas of the ventricular ependymal surface which warranted further follow-up (Figure 8).

At the age of one year and three months, circumcision and release of a tight frenulum associated with a mega meatus was done, and it went uncomplicated. At the age of one year and seven months, he was admitted again for another episode of acute bronchiolitis which was managed according to standard recommendations. However, two months later, at the age of one year and nine months, he was readmitted for pneumonia management.

At the age of two years, the patient encountered gastroenteritis and was admitted for rehydration and fluid management. At the age of two years and four months, closed reduction of a left femur fracture with hip spica application was performed which went uncomplicated. A month later, at the age of two years and six months, he was admitted again due to bronchopneumonia and managed with intravenous antibiotics. His FTT, global developmental delay, and characteristic facial features, including an elongated face with frontal bossing, suggested Russell-Silver Syndrome, prompting a targeted gene study that returned negative results.

**Figure 9:**
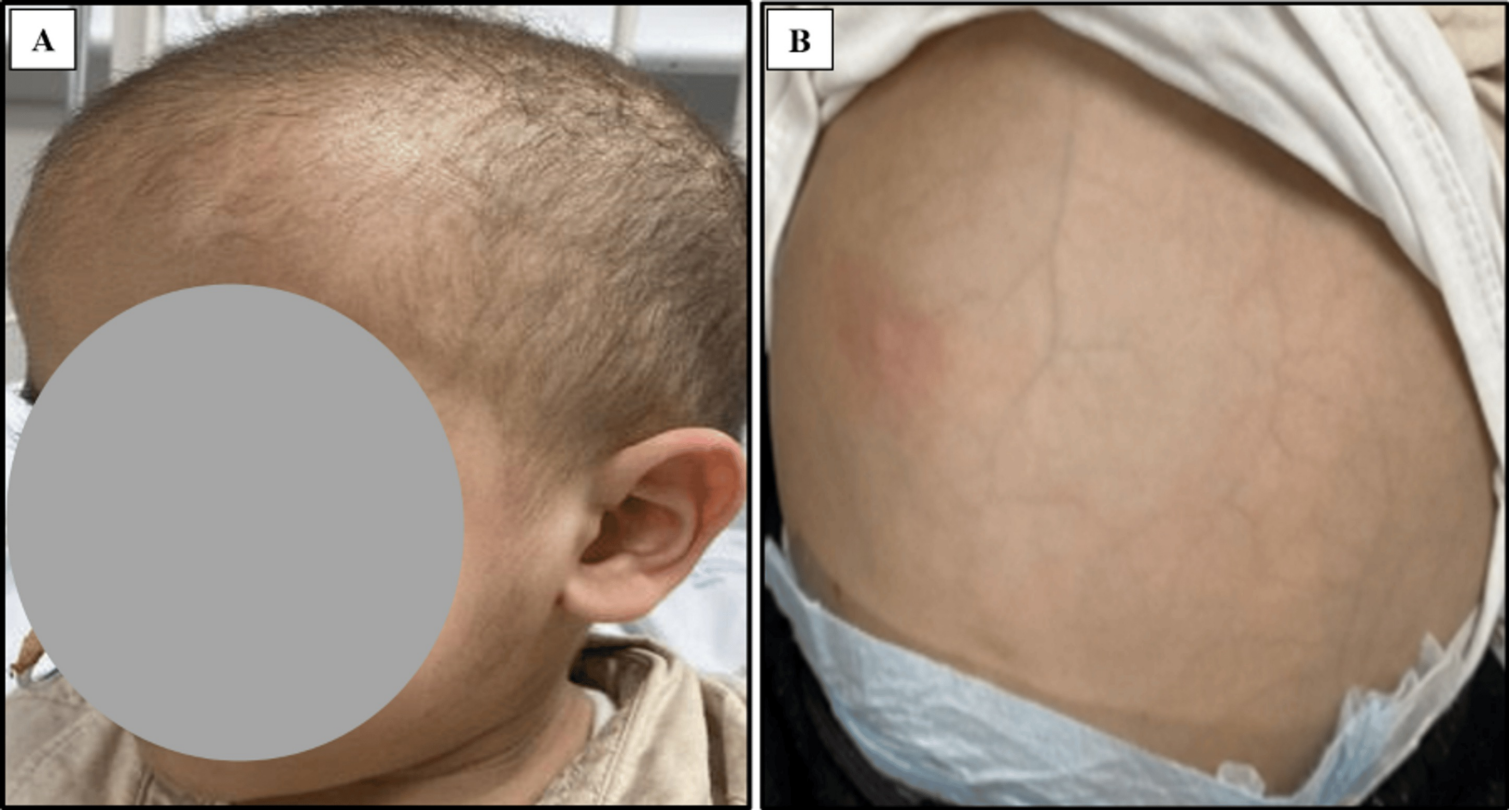
Patient 2 with trichohepatoentric syndrome type 1 The patient's pictures show macrocephaly with narrow elongated face, frontal bossing, and fair hair (A) associated with abdominal distention (B)

At the age of three years, he was admitted for salmonella gastroenteritis. On examination, cardiovascular and respiratory systems were normal. Neurological examination detected hypotonia but normal deep tendon reflexes. The patient exhibited brachydactyly, frontal bossing, relative macrocephaly, a narrow-elongated face, and fair hair (Figure 9A) associated with distended but soft and lax abdomen with no organomegaly (Figure 9B).

Developmentally, the patient walked at two and a half years, and he could only say "mama" and "baba," indicating a delay in milestones. Notably, his clinical condition was resolved with standard treatments, and further investigations were not deemed necessary.

At the age of three years and four months, he was diagnosed with hypoalbuminemia secondary to protein-energy malnutrition and protein-losing enteropathy. Laboratory workup (Table 1) revealed normal liver function tests, apart from hypoalbuminemia and elevated serum globulin, with normal electrolytes levels and renal functions. The patient's complete blood count showed leukocytosis and thrombocytosis. Additionally, at the age of three years and nine months, immunological tests were done and revealed significant increase in T-lymphocytes, with CD8^+^ cytotoxic T-cells being higher than CD4^+^ helper T-cells. Following repeated admissions and recurrent diarrhea, whole-exome sequencing was considered, which identified two homozygous variants of uncertain significance in the TTC37 gene, correlating with THES type 1.

Currently, he is four years old, his weight is 9.3 kg, and his height is 81 cm, both of which fall below the third centile for his age and sex (Figure 7). He is following up with multidisciplinary teams including endocrinology regarding short stature, otorhinolaryngology regarding speech, dietitian for feeding adjustment, and neurology. Recent computed tomography of the brain was performed under sedation as a follow-up of the previous MRI findings, and it showed no anomalies. The patient's primary diet consists of Peptamen Junior, an easy-to-digest and absorb formula, along with blended foods (potato, nuts, and fruits) administered through the NGT. This reflects poor oral intake and ongoing FTT. Due to these nutritional challenges, he is expected to require TPN in the near future.

## Discussion

**Figure 10:**
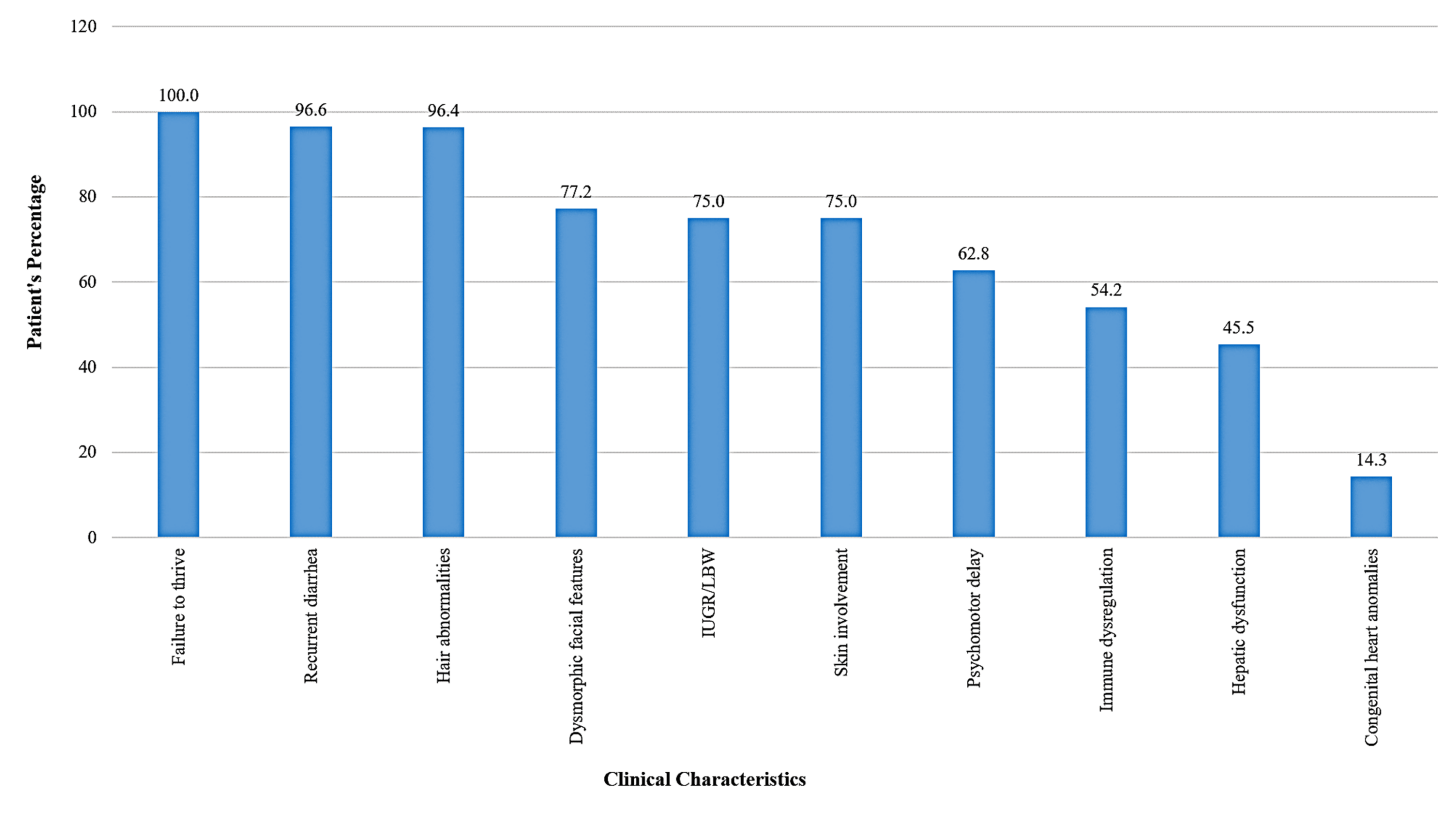
Clinical features of patients with trichohepatoenteric syndrome IUGR: intrauterine growth restriction; LBW: low birth weight Summary of 58 previously reported cases including the current report [5-19]. Image credits: Hasan M. Isa and Ghufran J. Ali

This report presents the first two children with THES in Bahrain, contributing to the globally reported 58 cases of this rare disease [5]. Based on 58 previously reported cases including our cases [5-19], the clinical characteristics of patients with THES are summarized in Figure 10.

This disease was primarily described in European and Asian countries, including neighboring countries to Bahrain such as Saudi Arabia [5]. Most of the studies are either case reports or case series which further highlights the condition’s rarity and the associated diagnostic challenges.

Recurrent intractable diarrhea is one of the most common presenting symptoms and is considered the initial symptom of THES [1]. This was the case in both of our patients. Similarly, recurrent diarrhea was present in all 48 cases reviewed by Fabre et al. [1] as well as the cohort study of 30 patients by Alsaleem et al. [5]. However, recurrent diarrhea was not reported in Poulton et al.’s [11] and Kinnear et al.’s [17] case reports involving an eight-year-old girl and a three-month-old boy, respectively, where the patients did not complain of recurrent diarrhea but rather presented with other clinical symptoms of genetically confirmed THES. In THES, diarrhea typically begins in the first week of life but may be delayed up to one year [1]. This is consistent with our patients, where diarrhea started at three months in Patient 1 and at seven months in Patient 2.

Hair abnormalities are another striking feature of THES and were found to be present in both of our patients. These often present as easily detached, woolly or hypopigmented hair [1,2]. Stankler et al. [3] identified characteristic microscopic features of brittle hair, such as woolly, thin strands, along with deficiencies in certain amino acids, particularly sulfur. In the Fabre et al.’s [1] review, hair abnormalities were present in all 48 cases, while Alsaleem et al. [5] reported them in 96% of their cases (n = 28/29). However, Vardi et al. [13] reported a four-month-old female baby who did not have hair abnormalities, which was attributed to her having short hair at the time of examination.

Dysmorphic facial features are another presenting sign found in both of our patients including frontal bossing with a large forehead, wide nasal bridge, and widely separated eyes. In Fabre et al.’s [1] review, dysmorphic facial features were present in all patients (n = 47/47), while Alsaleem et al. [5] reported facial dysmorphism in 73% of cases (n = 22/30). Though, Vardi et al. [13] and Lee et al. [15] reported four-month-old and five-month-old female patients, respectively, whom dysmorphic facial features were not prominent. They attributed this finding to the patients’ young age, and they suggested the possibility that facial features may become more apparent as the child grows.

The majority of patients with THES have a history of IUGR and low birth weight (LBW), followed by FTT later on, as reported in our cases. Supporting this finding, Fabre et al. [1] noted that 31 out of 46 cases (67.3%) had a history of IUGR. However, IUGR was not reported in Lee et al.’s [15] and Mahjoub et al.’s [16] case reports involving one-month-old and three-year-old male patients, respectively. In addition, Alsaleem et al. [5] reported that 20 (66.7%) of their 30 cases having a background history of LBW and all the 30 patients developed FTT. FTT was noted in all the reviewed patients as shown in Figure 10. Moreover, most patients present with short stature later in life [1,4]. In THES, FTT is mainly attributed to chronic diarrhea which disturbs nutrients absorption and causes significant nutritional challenges often necessitating supportive parental nutrition [20].

Both of our patients presented with recurrent infections necessitating admission, which might indicate immune dysregulation. In our patients, immune tests showed low IgG levels and low B-cells in Patient 1 with T-lymphocytosis in both patients. Supporting this, Fabre et al. [1] mentioned that immune dysregulation can have various presentations; it may manifest as recurrent infections or decreased levels or dysfunction of immunoglobulins and immune cells. Some patients may show diminished response and antibody production following vaccine administration [1]. Immune dysregulation was noted in 39 out of 44 cases (88.6%) according to Fabre et al.’s [1] review. Moreover, the vast majority of cases presented in Figure 10 demonstrated this condition, except for Alsaleem et al.’s [5] cohort where it was observed only in five out of 24 cases (21%), while three cases reported by Albar et al. [10], Xinias et al. [12], and Dweikat et al. [18] had no immune dysregulation.

Hepatomegaly and liver enzyme dysregulation were present only in Patient 1, while Patient 2 had no liver involvement. This finding aligns with Fabre et al.’s [1] review where most of the patients (n = 22/33, 66.6%) presented with liver disease, manifesting as hepatomegaly, cirrhosis, or hemosiderosis. Moreover, Alsaleem et al. [5] reported chronic liver disease in nine out of their 30 cases (30%).

Skin abnormalities in THES can present as café au lait patches, excessive dryness, or elastic skin [1]. In our report, Patient 1 presented with café au lait spots and dry skin, while Patient 2 had no skin manifestations. Alsaleem et al. [5] reported skin abnormalities in 86.7% of patients, while in Fabre et al.’s [1] review, skin manifestations were noted in half of the patients (50%).

Other rare findings associated with THES include congenital heart diseases that could present as septal anomalies or large vessel abnormalities [1]. Cardiac anomalies were found to be present in 25.8% of cases reviewed by Fabre et al. [1] and only in 3.3% of patients studied by Alsaleem et al. [5]. In our report, Patient 1 had a cardiac anomaly in the form of ASD. This is similar to cases reported by Alrammal et al. [7], Xinias et al. [12], and Lee et al. [15]. Additionally, ventricular septal defect was detected in cases reported by Alrammal et al. [7] and Poulton et al. [11]. Lee et al. [15] had also reported coarctation of the aorta in one of his cases, while Vardi et al. [13] reported patent foramen ovale.

Patient 1 presented with a bilateral inguinal hernia which is another rare feature of THES. Alrammal et al. [7] and Dweikat et al. [18] also reported inguinal hernia in their two-year-old and ten-month-old female patients, respectively. However, inguinal hernia could be a coincidental presentation in THES patients [1]. Platelet abnormalities manifesting as enlargement in size, kidney anomalies, and thymus dystrophy are additional rare features that have been reported in a few cases as well [1]. However, none of our cases presented with these symptoms.

Psychomotor delay had also been documented [1,5]. Both of our cases demonstrated delays in motor and mental development. Alsaleem et al. [5] found that 57% of their cases exhibited some form of psychomotor delay. While Fabre et al. [1] noted that mild forms of mental impairment are present in more than half of cases. In other instances, THES may present with motor delay only, as seen in Lee et al.’s [15] and Mahjoub et al.'s [16] case reports involving five-month-old and one-month-old female patients, respectively.

The phenotype-genotype of this disease is not well understood owing to the disease rarity and the genetic variations [1,21]. Patient 1 exhibited more severe symptoms characterized by chronic diarrhea, hair abnormalities, liver impairment, higher frequency of infections and admissions, congenital heart disease, and inguinal hernia compared to Patient 2 who had milder symptoms. This difference could be potentially attributed to the presence of three homozygous gene mutations in Patient 1 compared to two mutations found in Patient 2.

The clinical features of THES can overlap with various other diseases and syndromes. For instance, Russell-Silver Syndrome was initially suspected in Patient 2 due to distinct facial characteristics and developmental delay but was excluded through genetic testing. Other differential diagnoses for THES include various causes of diarrhea such as infectious and allergic etiologies which can be ruled out through thorough investigations [1]. Malnutrition also presents as a potential differential diagnosis affecting hair color and shape; however, symptoms typically improve with appropriate nutritional intervention [1]. Café au lait patches may raise suspicion for neurofibromatosis but can be excluded through clinical assessment [1]. Congenital tufting enteropathy is another autosomal recessive disorder sharing a similar presentation but characterized by intestinal tufts [2].

Similar to all the reviewed studies, the diagnosis of our two patients with THES primarily relied on genetic testing. Upon genetic testing, three different variants associated with TTC37 were detected. The first variant, c.2170T>C (p.Cys724Arg), was detected in homozygosity. It causes an amino acid change from cysteine to arginine at position 724 in exon number 21. It is a novel mutation that has not been reported earlier and not reported in ClinVar. However, according to in silico parameters, it is predicted to be damaging and disease-causing and has an extremely low frequency in gnomAD population databases [22]. It has been observed in both of our patients. The second variant, c.4507C>T (p.Arg1503Cys), causes an amino acid change from arginine to cysteine at position 1503 in exon number 42. According to the Human Gene Mutation Database (HGMD) Professional 2022.4, this variant has previously been described as disease-causing for THES by Kinnear et al. [17]. ClinVar lists this variant as having conflicting interpretations of pathogenicity, uncertain significance (4), and pathogenic (1), with variation identification number (ID): 287653 [23]. It is also classified as a variant of uncertain significance according to the American College of Medical Genetic and Genomics (ACMG) standards [24,25]. According to in silico parameters, it is predicted to be damaging and disease-causing and has an extremely low frequency in all the gnomAD population databases [25]. This variant was also detected in both affected patients. The third variant, which was detected in homozygosity, c.4534C>T (p.Pro1512Ser), has not been reported earlier. It causes an amino acid change from proline to serine at position 1512 in exon number 42. ClinVar lists this variant as having conflicting interpretations of pathogenicity, uncertain significance (2), and likely benign (1), with variation ID: 546976 [26]. According to the ACMG guidelines, it is considered to be a variant of uncertain significance [24,27]. In the in silico parameters, it is predicted to be benign, not damaging [27]. This variant has not been detected in Patient 2.

THES is a complex genetic disorder predominantly caused by mutations in the SKIV2L and TTC37 genes, which are crucial for mRNA processing and normal cellular functions [1,2]. These genes encode proteins similar to yeast Ski2p and Ski3p, essential components of the Ski complex involved in RNA regulation [1,14,28]. Fabre et al. [2] indicated that 31% of cases were linked to mutations in the SKIV2L gene (SKIC2), while 69% were associated with mutations in the TTC37 gene (SKIC3). Notably, both of our patients fell into the latter category. Bourgeois et al. [20] suggested that SKIV2L gene mutations typically result in more severe symptoms, particularly regarding growth parameters and liver damage. Although both of our patients had TTC37 gene mutation, they also presented with severe symptoms, mainly Patient 1. While genetic testing for SKIV2L and TTC37 mutations is essential for diagnosis, accessibility issues in developing countries significantly hinder this process, posing substantial challenges for diagnosis and management.

Management of THES remains challenging and is often focused on supportive treatment including mainly feeding support and treatment of infections. Patients with THES often require parenteral nutrition with high-energy formulae [29]. In our patients, supportive care regarding nutrition was prioritized. Patient 1 received extensively hydrolyzed milk formula with high-energy formula via NGT, TPN, followed by transitioning to solid food. Patient 2 also utilized NGT feeding along with solids introduction. Due to persistent diarrhea, nearly all patients eventually require TPN [1,20,28]. Fabre et al. [29] demonstrated that patients requiring TPN were typically weaned off after approximately 15 months.

Infection management is challenging in THES, with many patients requiring regular immunoglobulin supplementation [29]. In our patients, respiratory and gastrointestinal infections were managed according to the recommended antibiotics. Moreover, Patient 1 required immunoglobulin supplementation due to recurrent infections. However, Girault et al. [4] explored antibiotic treatments for diarrhea but found no significant effectiveness. Steroids and immunosuppressants have been tried with only limited success when combining cyclosporine and steroids [4]. Bone marrow transplantation has also been explored; unfortunately, one patient died due to severe interstitial pneumonia posttransplant [4].

On follow-up, both of our patients showed a gradual increase in their weight and a reduction in the severity of diarrhea. However, they remained below the third centile for weight and height. Similarly, Zhang et al. [9] reported a one-and-half-month-old female patient with weight and height below the third centile for age and sex, while Poulton et al. [11] reported an eight-year-old female patient with weight and height below the first centile for age and sex. The prognosis for THES is multifactorial because many patients face complications such as organ failure and infections that can be fatal [1,14].

In this report, we encountered several limitations. First, both of our patients presented with TTC37 gene variants which limited the comparison of the findings regarding the potential differences in clinical manifestations between TTC37 and SKIV2L variants. Additionally, this report was conducted at a single hospital in Bahrain, which may not fully represent the broader population. Furthermore, some data were missing in Patient 2 related to the use of immunoglobulins and the exact degree of relationship between the two patients’ families. Despite these limitations, the findings of this report are important and fill the gap of knowledge about this rare disease in this geographical region. 

## Conclusions

THES is a rare autosomal recessive disorder characterized by a triad of hair abnormalities and dysmorphic features, liver disease, and intestinal dysfunction, primarily manifesting as severe diarrhea which could be the first clue to this syndrome. This condition is often associated with a history of IUGR and significant FTT. A better understanding of this syndrome and timely diagnosis through genetic testing could play a crucial role in managing associated complications and improving prognosis. However, further studies are required to clarify the phenotype-genotype correlation, assessing long-term outcomes, and innovative therapeutic options such as gene therapy. Moreover, involving multiple centers and a larger cohort would be beneficial to validate and expand upon our findings.
